# Robodeport or surveillance fantasy?: how automated is automatic visa cancellation in Australia?

**DOI:** 10.3389/fsoc.2024.1336160

**Published:** 2024-04-10

**Authors:** Leanne Weber, Alison Gerard

**Affiliations:** Canberra Law School, University of Canberra, Canberra, ACT, Australia

**Keywords:** deportation, non-citizens, automation, visa cancellation, automated governance, criminal justice technologies

## Abstract

Australia has been widely condemned for its harsh and comprehensive external border controls that seek to control the inward mobility of would-be asylum seekers through visa denial, interdiction and offshore detention. Less widely discussed is the fact that internal controls have been repeatedly ramped up over the past two decades. This includes the administrative removal of lawfully-present non-citizens following visa cancellation on character grounds under s501 of the Migration Act 1958 (Cth). Automatic visa cancellation was introduced in 2014 for non-citizens sentenced to a prison term of 12  months or more, or for certain offences, bypassing individualised decision-making and raising the spectre of a visa cancellation pipeline feeding a highly automated deportation machinery. In an age of increasingly automated forms of governance, a key question that arises is the role that has been played by automated systems in achieving what has been a seismic shift in practice, and the normative implications of any developments towards automation within the visa cancellation and removal systems. This paper outlines the shift towards automation in other systems of governance in Australia—most notably the notorious Robodebt scheme—before examining automation in Australia’s visa cancellation system. Documentary analysis of recent parliamentary inquiries, independent reports and government policy is used to piece together the development of inter-agency data exchange practices and automation over three specific periods—historical practice pre-2014, post-2014 to the present, and proposed future developments. We conclude that Australia’s s501 visa cancellation system is neither automated nor automatic. Rather, the 2014 law reform gave rise to a ‘surveillance fantasy’ with immense consequences for non-citizens, particularly those who face long days in immigration detention at the conclusion of their prison sentence. We show that while concerns about increasing automation are well-founded, systems based on less sophisticated forms of information handling and reliant on human decision-making nevertheless continue to raise age-old questions concerning efficiency, accuracy and fairness.

## Introduction: what is automatic visa cancellation?

Australia has been widely condemned for its harsh and comprehensive external border controls that seek to control the *inward* mobility of would-be asylum seekers through visa denial, interdiction and offshore detention. Less widely discussed is the fact that internal controls have been repeatedly ramped up over the past two decades. This includes the administrative removal of lawfully-present non-citizens following visa cancellation on character grounds under s501 of the *Migration Act 1958* (Cth).^1^ In this case, state power is used to generate, rather than prevent, border crossing through expulsion to countries of citizenship. In this article we aim to problematize this use of state power to enforce outward mobility, with an emphasis on the systems used to achieve this exclusionary outcome.

The provision used most often to generate this outward mobility is contained in s501(6)(a) which authorises cancellation of visas for non-citizens held in prison who are deemed to have a ‘substantial criminal record’. ^2^ For the first time, legislative changes in 2014—apparently modelled on UK practices—mandated *automatic* visa cancellation by inserting s501(3A) of the *Migration Act 1958* (Cth). This amendment means that a visa *must* be cancelled if a non-citizen receives a prison term of 12 months or more, or is convicted of sexually based offences involving a child, and is serving a sentence of imprisonment on a full-time basis.^3^ The automatic cancellation provisions were designed to contain non-citizens liable to visa cancellation so that they were not released into the community at the end of their prison term but instead transferred to immigration detention. As such, the reforms increased the importance of identifying non-citizens potentially liable for visa cancellation within prisons, so that administrative powers could be used to immobilise them in preparation for removal.

On the face of it, the effect of the legislative change was to enable the cancelling of a visa before any consideration of individual circumstances, such as family connections in Australia, length of residence and likely future risk to the Australian community. Automatic visa cancellation decisions are routinely delegated to public servants from the National Character Consideration Centre (NCCC), a part of the department that specialises in s501 cancellations. This specialist centre was created even before the 2014 legislative reforms, as maximising the discretionary use of s501provisions had already become a government priority. Natural justice, the notion of procedural fairness that operates to protect against bias and limit government decision-making powers, would thereafter only be considered after a visa is cancelled under the automatic provisions. Natural justice has been progressively weakened in Australia’s migration control system and results in a distancing of non-citizens from decisions and decision-makers (Elton, 2022).

**Figure 1:**
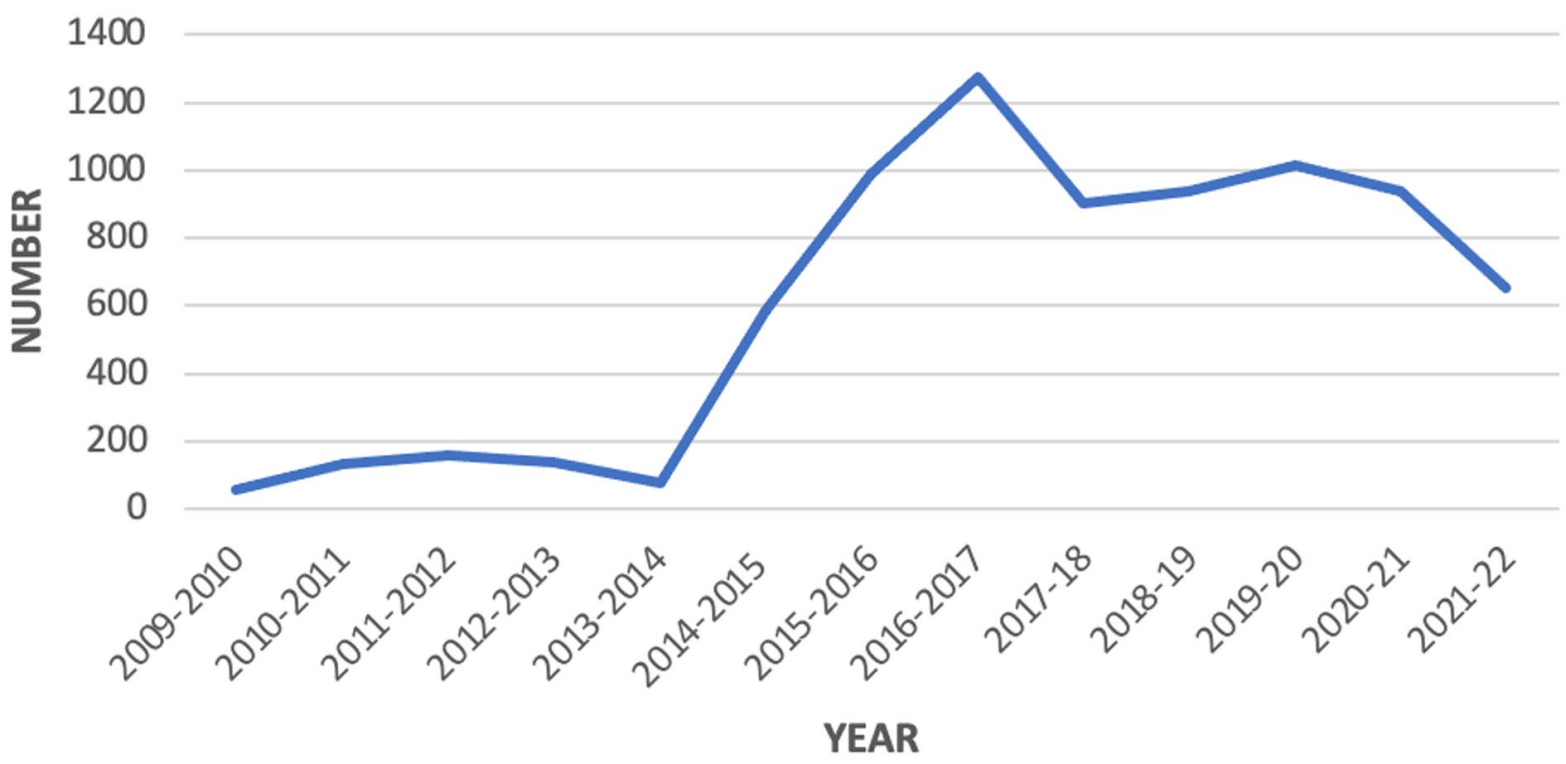
s501 visa cancellations 2009/10–2021/22. Source data extracted from Dept of Home Affairs annual reports and visa cancellation statistics website.

It must be said that discretionary decision-making prior to 2014 was already tilted heavily in favour of cancellation, since the influence of mitigating factors specified in highly prescriptive Ministerial Directions that guide both departmental^4^ decisions and external appeals to the Administrative Appeals Tribunal had already been progressively reduced (Weber and Powell, 2020; Powell and Wickes, 2023), elevating administrative efficiency over questions of procedural fairness. For example, even long-term residence does not provide immunity from s501 visa cancellation, as it did with earlier cancellation regimes. Even so, what followed the 2014 reform was a spectacular rise in the number of visas cancelled under s501 from 76 in 2013–14 to a peak of 1,277 in 2016–17, with high levels of cancellations sustained for several years thereafter (see Figure 1).

The introduction of automatic cancellations had produced an instant and enduring effect on the state’s capacity to expel non-citizens which impacted long term residents, recognised refugees and asylum seekers alike. So significant were the effects that it might be considered an act of re-bordering, driven by rising nationalistic sentiments, overblown fears about community safety and contestation over membership (Stumpf, 2006). In addition to determining *who* would be removed, these changes had the potential to bring changes in *how* this would be achieved, raising the spectre of a visa cancellation pipeline feeding a highly efficient removal machinery that was potentially denuded of human consideration or contact. In an age of increasingly automated forms of governance, where technology is often seen as the solution to problems of administrative efficiency, key questions arise about the role that has been played by automated systems in achieving this seismic shift in practice, and the normative implications of any developments towards automated decision-making within the visa cancellation and removal systems.

At this point it may help to define how some key terms are being used in this article and to clarify the distinction made throughout between *automatic* and *automated* processes. *Automatic* visa cancellation^5^ refers to a *legal requirement* for mandatory cancellation that removes discretion from human decision-makers and omits considerations of natural justice. This concept is agnostic with respect to the way in which the instruction is implemented and can produce significant increases in the rate of expulsion even without sophisticated technology. *Automated* governance, on the other hand, refers to the *technologies* employed to administer a particular policy or legislative requirement. It did not become apparent to us until the analysis had progressed, that the terms mandatory and automatic, which we believed initially to be inter-changeable, are also distinguishable in important ways that are explored towards the end of the article.

Reliance on techniques that bypass human judgement to identify potentially removable non-citizens, might seem to avoid some of the inconsistency, bias and other pitfalls associated with human decision making (Hoang and Reich, 2017). However, the increased use of automated systems carries normative implications of its own with respect to the removal or displacement of human discretion within human-non-human networks. In the next section we provide a brief overview of the emergence of automated governance and identify some of the elements that may be present within an automated decision-making system.

## Developments in automated governance

With rapid developments in both globalisation and information technology, policing and other forms of governance increasingly operate in ‘informated spaces’ (Sheptycki, 1995). Developments in the use of technology over the past few decades can also be described in terms of digital or e-government. So ubiquitous are these changes that the United Nations has developed an explicit e-government strategy in which Australia features as one of the world’s leading exemplars (United Nations, 2022; see Table 1). As Chung (2020, p. 8) notes, the term has been employed to mean everything from ‘online government services’ to ‘exchange of information and services electronically with citizens, businesses, and other arms of government’. Broadly speaking, any technological system aimed at ‘achieving public ends by digital means’ (p. 8) could be considered an example of e-government. While the term is most often applied to the provision of services, it could equally be applied to law enforcement regimes.

Data sharing between government agencies often forms the ‘bedrock’ for digital government (Zhou et al., 2021, p. 1286). While the language used around technological developments often conveys an impression of unimpeded integration across electronic systems, the reality is often otherwise, and the smooth flow of data between agencies is far from straightforward or guaranteed. Zhou et al. suggest it is more common for data to ‘stick like glue’ than to ‘flow like oil’ across digital systems, due to a variety of barriers to information exchange, and note that the development of ever more sophisticated technologies is rarely the solution to identified problems of governance.

Similar shortfalls in digital governance have been noted within policing and migration control systems. Sheptycki (1995) has argued that information exchange systems that are central to transnational policing in Europe typically arose from existing legal and organisational structures, were cobbled together in piecemeal fashion through multiple bilateral agreements, and could not be considered to constitute an ‘integrated informated space’. Some years later, when Aas (2011) analysed border control databases being developed across Europe in response to the development of the Schengen ‘free movement’ zone, she still found that the ‘crimmigration assemblages’^6^ involved in cross border data exchange were fragmented and permeable, and better characterised as ‘surveillance fantasies’ than as watertight surveillance systems. Despite persistent shortfalls, European governments have continued to securitize their borders using ‘technologically induced solution[s]’ (Bigo et al., 2020). This is apparent in ongoing efforts to increase ‘interoperability’ (i.e., the capacity for one user to search across multiple databases simultaneously) in pursuit of efficiency dividends that, according to Bigo et al., are yet to materialise.

While these examples concern cross-border information exchange, Zhou et al. (2021) also identified considerable challenges in data exchange between agencies at different levels of government within China, with inter-relationships between agencies proving to be paramount. This is an important consideration for internal border control systems in a federated country such as Australia where information exchange between federal immigration authorities and criminal justice agencies operated by states and territories is crucial.^7^ For example, in previous research into ‘migration policing networks’ in one Australian state (Weber, 2013), state or federal government agencies that agreed to provide data or cooperate in other ways with immigration authorities generally did so only where this satisfied some pre-existing organisational objective unrelated to border control, rather than through complete identification with border control objectives.^8^

While governments tend to concentrate on increasing the accuracy, efficiency and connectivity of digitised information systems, other commentators look beyond this technological obsession. Bigo et al. (2020) note that framing problems of governance in purely technical terms depoliticises them and disguises urgent human rights concerns about data sharing, privacy and fairness, such as those that arise from the quest for interoperability across European borders. And, as Parmar (2019) points out, new digital technologies and information exchange used in the policing of internal borders can give the illusion of efficiency and neutrality while systematically targeting racialised populations for stratification and exclusion.

Beyond these examples of digital or e-govern*ment* comprising automated data exchange, governments around the world are now embracing what Chung (2020) calls ‘digital govern*ance*’. This involves automated decision making, the use of predictive algorithms and generative AI, which signals the onset of an ‘*intelligent* information age’ considered by some analysts to constitute a ‘Fourth Industrial Revolution’. One example within law enforcement is the application of algorithmic techniques in risk assessment using ‘big data analytics’ (Hannah-Moffat, 2019). The embedding of technologies into systems of decision making that were once operated entirely by human actors has given rise to a growing literature on hybrid human-non-human networks (or human-thing assemblages) in which ‘actants’ (a term derived from Actor Network Theory) are regarded as sources of action regardless of their human or non-human status (Milivojevic, 2021). That literature cannot be reviewed fully here, but one point worth noting is that unpredictability may be built into even highly automated systems via ‘mediators’ that ‘can make something happen that is not necessarily an outcome of what is set in motion by actors/actants in the network’ (Milivojevic, 2021, p. 26). In other words, even highly automated systems may not produce identical outcomes in all comparable circumstances.

Some instances of automation already exist in the Australian law enforcement context. One example is the widely used speed camera system referred to by O’Malley (2010) as ‘telemetric policing’ because of the total lack of contact between policed persons and the police. Fine notices are automatically generated on the basis of electronically captured data and despatched to the imputed driver on the basis of vehicle registration information, an effect O’Malley (2010, p. 805) refers to as ‘simply a matter of one machine “talking” to another’. Individuals caught up within such codified systems are effectively transformed into units of digital risk information or ‘data doubles’ (Haggerty and Ericson, 2000, p. 605), stripped of their *in*dividuality and becoming what Deleuze has described as ‘dividuals’ (cited in O’Malley, 2010, p. 796). In other words, digitization effectively empties the official record of human content, with significant implications for individualised justice: ‘[whereas] *in*dividuals are the bearers of rights and create political costs; dividuals simply have to be registered and coded’ (O’Malley, 2010, p. 796, emphasis added).

Traditional modes of enforcement often rely on the active *dehumanisation* of those subject to particularly punitive measures and intense surveillance through demonising public rhetoric. In addition, the subject of automated law enforcement may be marked for harsh intervention by more routine and less detectable processes that *neutralise* their humanity, through the emptying out process discussed above. Whereas dehumanisation is infused with negative emotion, neutralisation is the erasure of positive emotion through distancing. It is important to note that neutralising effects may also be produced without the use of advanced technologies, via *emotional distancing* associated with any large bureaucratic system that emphasises standardisation and efficiency and separates decision-makers from the subjects of their decisions (Weber, 2005).

Bosworth and Guild (2008, p. 709) have observed a similar trend across the migration control system in Britain, fuelled by managerialism and the quest for administrative efficiency: ‘Rather than being treated as individuals with specific needs and experiences, foreigners are instead grouped together and managed collectively as a matter of administrative expediency’. In the Australian context, Nicholls (2007, p. 149) notes that a different body of legislation initiating mandatory removal for unlawful entrants in 1989 – which preceded the s501 mandatory visa cancellation measures by 25 years—turned administrative removal into a ‘well-oiled machine’, enhancing the onus on law enforcement officials to detect unlawful non-citizens. And with respect to visa cancellations in Australia, Nethery (2012, p. 735) concluded that ‘mechanical’ decision-making using fixed criteria, leads to visa cancellations made ‘without the checks and balances usually associated with administrative decisions’. While bureaucratic decision-making systems may already create a distancing effect, automated governance can intensify these pre-existing concerns or give rise to new fears about fairness and accountability.

One challenge raised by Milivojevic (2021, p. 27) in relation to algorithmic systems is the effect of ‘blackboxing’ in which ‘[w]hat is in the black box no longer needs to be considered and cannot be problematised (Michael, 2016); inputs and outputs are the only points we need to discern’. Where politicians and administrators consider automated systems to be inherently reliable, if not infallible, there is a noticeable reluctance to look within the ‘black box’. Indeed, referring to ‘techno-solutionist’ approaches at Europe’s external borders, Forti (2024) has concluded that legal frameworks are totally inadequate to address any algorithmic errors that may occur in such opaque, AI-driven systems. Furthermore, reliance on operational data collected by police to feed into these systems may amplify and ‘hardwire’ pre-existing discrimination within policing practice: ‘The belief in the independence and objectivity of data-driven policing solutions and, in particular, predictive policing programmes will send law enforcement officers to monitor and detect crimes in the same already over-policed communities’ (Williams and Kind, 2019, p. 15; see also Ugwudike, 2020). As McGuire and Renaud (2023, p.456) conclude, in relation to the disastrous use of algorithmic auditing tools by the UK Post Office, ‘our very capacity to conceptualise harm is being reshaped by the imperatives of technologies’.

Depending on the distribution of power between the human and non-human elements, so-called techno-social assemblages may also shrink the space for the exercise of discretion in the name of individualised justice. This raises the possibility that ‘[o]nce humans are removed from this [algorithmic] process, which is a possibility, protections of human rights and civil liberties will entirely be dependent on smart things’ (Milivojevic, 2021, p. 31). It is important not to exaggerate the ‘agentic importance’ of non-human elements in hybrid systems and to note that automated systems may have some inbuilt capacities to recognise extenuating circumstances. Still, it is often the case that individuals may only come into view as fully human where they have opportunities to challenge automated decisions such as the issue of a system-generated fine or, more pertinent to this discussion, an automatic visa cancellation decision in person. As O’Malley (2010, p. 805) explains: ‘At this point we shift from the simulated anonymity of the world of machines and codes into the realm of living agents and disciplinary institutions.’

This brief review of some of the literature on automated governance has identified several distinct components that contribute to automated decision making. *Electronic data sharing* across government departments and sometimes state or national borders may be a prelude to more advanced forms of e-governance. Digitised data then lends itself to *automated data matching* that seeks to identify records occurring in two or more files, as observed earlier, solely via ‘one machine “talking” to another’, thereby obviating the need for human operators to cross check information on a case-by-case basis. A relevant example for this discussion might be a system that applied an electronic protocol to produce a list of individuals who were named in prison admission files and were recorded as non-citizens by immigration authorities.

A more sophisticated form of automated governance might involve *algorithmic decision-making* in which categorisations and predictions based on pre-determined criteria, and/or judgements about courses of action to follow from these identification processes, are made—at least in the first instance—without human intervention. A hypothetical example relevant to this discussion would be if decisions to cancel visas were produced entirely by non-human decision makers.^9^ Human-non-human systems of governance may reflect differing degrees of automation and human intervention depending on which of these steps are enacted and the broader organisational protocols in which they are embedded. In the remaining discussion we aim to identify the balance between human and non-human ‘actants’ in the s501 visa cancellation system and consider the normative implications arising from current, and possible future, practices.

## From Robodebt to Robodeport?

Before considering the empirical evidence for automation in the s501 visa cancellation system, this section sets out our expectations as we began this investigation and provides further background and comparative information about the evolution of the system in Australia. Automatic deportation was introduced in the UK from 2007 for convicted non-citizens serving prison sentences of 12 months or more (Bosworth, 2011; Aliverti, 2013). Prior to that, deportation was discretionary and could follow a Ministerial decision that expulsion was ‘conducive to the public good’, or a court recommendation associated with a criminal conviction.^10^ However, after intensive empirical research, the process of *automatic* visa cancellation in the UK was found to be anything but *automated*, instead necessitating intrusive and discriminatory questioning on admission to prison, aimed at the identification of potentially deportable non-nationals (Bosworth, 2011; Kaufman, 2012). At least in the early stages of policy implementation, the resulting information was provided by prisons to immigration authorities via fax (Bosworth, 2011). Several years later, after the policy of concentrating deportable inmates into selected ‘hubs and spokes’ facilities had been introduced, Kaufman (2012, p. 701) still described the process of identifying deportable prisoners as ‘piecemeal’, based on ‘racialised assumptions about foreignness and British national belonging’ and often resulting in inaccuracies arising from misidentifications.

There are several reasons to expect that the automatic visa cancellation system introduced in Australia some 7 years after the British system might be more technologically advanced. Firstly, previous research into ‘migration policing networks’ in Australia conducted by one of the authors more than a decade ago found that agreements for electronic data exchange and cross-agency data matching were already well developed within parts of the migration control apparatus concerned with detection of immigration infringements. This included numerous formal and informal agreements between immigration authorities, the Australian Tax Office, educational institutions, hospitals, local councils, the federal social security agency Centrelink, transport authorities and regulatory bodies, to facilitate the location of ‘unlawful non-citizens’ (Weber, 2013). Information sharing between immigration authorities and state and federal police was also on the increase at that time, primarily through case-based enquiries that were mediated by immigration staff posted within police organisations, but also with reference to a specialist database operated by immigration authorities (the Immigration Status System or ISS) that was designed to provide up to date information on visa and citizenship status to law enforcement officials (Weber, 2011). While the accuracy of ISS information has been questioned (see below) Australia’s universal visa requirement, operation of border controls at both entry and exit, and relatively few opportunities for clandestine entry, creates at least a theoretical possibility that the immigration status of every non-citizen present in Australian territory could be known and recorded there, providing a basis for electronically mediated data matching.

The question remains as to whether information exchange systems between immigration and criminal justice authorities *have* developed further since these earlier indications. Again, there are reasons to suspect they might have. The ISS system was a legacy of multiple external inquiries and internal reviews that followed a series of unlawful detentions, and in some cases removals, of Australian citizens based on racialized assumptions about nationality (Commonwealth Ombudsman, 2005, 2007; Foreign Affairs, Defence and Trade References Committee, 2005; Palmer, 2005). These practices were generally not related to visa cancellations on character grounds but have some implications for that cohort as well. Following the revelations about unlawful detention and removals, emphasis was placed by government, not on the fairness or otherwise of Australia’s strict visa regime and overly zealous removal machinery, but on the need for improved data systems to ensure these ‘mistakes’ did not occur again. Accuracy in the determination of identity and legal status became a paramount goal of migration control.

Remarkably, immigration officials were said to have been acting at times solely on the advice of other agencies, notably the police, without reference to their legal obligations and the need to verify the information provided. The solution to the profound unreliability and bias of this largely human-mediated system was seen to be via improvements in, and greater reliance on, information technology. This included the development of a ‘Strategic Plan for Identity Management in DIAC’ [Department of Immigration and Citizenship (DIAC), 2007, p. 4] and reliance on a new and improved ‘Integrated Case Management’ approach to guide decision-making, supported by an IT interface with the benevolent title of ‘Systems for People’ (Weber, 2013). Public communications around these developments at the time seemed to signal that a new age of technologically mediated migration control was dawning.

Another set of recent developments appears to reflect ongoing aspirations towards greater automation within the visa cancellation system; namely amendments to introduce ‘designated offences’ into the *Migration Act 1958* (Cth) that were proposed in 2018, 2019 and 2021 but failed to pass into law.^11^ Their introduction would have triggered discretionary visa cancellation, *irrespective* of sentences imposed by courts, in relation to a specific group of violent offences punishable by a maximum sentence of 2 years or more.^12^ Although the Explanatory Memorandum accompanying the Bills described the measures as providing ‘new, specific and objective’ grounds for visa cancellation,^13^ critics noted that the measures added no criteria for cancellation that were not already available (Visa Cancellation Working Group, 2021).

Rather, these proposed reforms seemed designed to send a message to the Australian public that further measures were being taken to protect them from ‘foreign criminals’, and to signal to judges in the criminal courts the political supremacy of immigration control. Addressing the designated offences provisions in Parliament, then Minister Alex Hawke noted: ‘I can provide examples to any member here of the judiciary over time in different courts—it might be the Victorian courts or the ACT Supreme Court—taking into account, in sentencing, the fact that this government will be deporting a person or cancelling their visa, with sentencing thereby coming in under the mandatory cancellation thresholds.’^14^ In response, the Law Council of Australia (2019, p. 6) suggested in a submission to a Senate Inquiry that the proposed legislation had ‘the potential to undermine the sentencing function of the judicial system and the discretion it possesses regarding sentencing offenders’.

Beyond this politico-juridical rivalry, the amendments, while seemingly redundant with respect to legal powers, might also be understood as a step towards a more automated administrative system. The Bill was reportedly designed to smooth the way for the more efficient functioning of removal. While the ‘new’ grounds for cancellation were discretionary, rather than automatic, their full realisation—had the amendments been passed—would have required, at minimum, greater formalisation of data sharing with immigration authorities concerning cases coming before the courts.^15^ Beyond this, and purely hypothetically, the removal from the decision-making equation of actual sentences imposed could open the way for a highly automated data matching system, requiring only that legislated penalties for the relevant offences in each state be kept up to date on a central system to assist in identifying eligible cases from court lists.

While doubts remain about the extent of technological advances actually achieved in the era of reform following the wrongful detention cases identified earlier, we know that highly automated systems of governance do exist in Australia in other federal policy areas. The clearest example is the notorious ‘Robodebt’^16^ scheme that deployed inter-departmental data matching and a now-discredited ‘income averaging’ algorithm to identify social security recipients who had allegedly mis-represented their earnings. As well as identifying supposed welfare fraudsters, the system automatically generated threatening letters to those identified by the algorithm requiring them to establish that departmental records (which they could not access) were incorrect, thereby reversing the usual onus of proof. A Royal Commission^17^ subsequently found the scheme to be both technically flawed and unlawful, and recognised the ‘cruelty’ and ‘disastrous effects’—including suicides—produced by an automated system that was totally devoid of human oversight. Among its 57 recommendations, the Royal Commission urged the Commonwealth to ‘consider establishing a body … with the power to monitor and audit automate[d] decision-making processes with regard to their technical aspects and their impact in respect of fairness, the avoiding of bias, and client usability’ (Rec 17.2).

While there are substantial grounds to speculate that the Australian migration control system may have been ahead of the game in terms of electronic data sharing compared with the UK, even before the automatic cancellation provisions were introduced, the question is far from settled. Our current study aims to determine the nature and extent of data exchange between immigration control and criminal justice authorities in Australia across a wide range of interactions related to s501 visa cancellation and removal.^18^ This article presents preliminary findings from one part of that study that shed some light on the methods of data sharing and levels of automation involved in the s501 visa cancellation process, drawing solely on documentary sources. Later stages of the research will include interviews with key government agencies that may provide more definitive answers about the data sharing and other forms of inter-agency cooperation that constitute the s501 visa cancellation system.

## How automated is automatic visa cancellation?

In order to investigate the extent of automation in Australia’s visa cancellation system, we relied upon documentary analysis of recent parliamentary inquiries, independent reports and government policy instructions focusing on criminal deportation and s501, including its operation and recent attempted, or actual, amendments. It is notoriously difficult to obtain information on the operation of Australia’s migration laws, practices and procedures due to the secrecy that cloaks this area of government operations. The government has previously introduced secrecy provisions, and for a long time, information regarding boat arrivals and the management of borders, externally and internally, has been pushed beyond the remit of public view. As a result, we have relied heavily on documentary research, including recourse to Freedom of Information (FOI) applications,^19^ to piece together this interrogation of the extent of automation in Australia’s visa cancellation and removal machinery.

A desktop review identified six major parliamentary inquiries that were within our remit. These included three from the Australian Senate’s Standing Committee on Legal and Constitutional Affairs and three Joint Parliamentary Committees.^20^ We developed several categories of agencies and key terms to code for in our examination of the submissions and final reports of these Public Inquiries. In terms of agencies, our key terms included both state and federal entities in Australia, noting that contentions arise between jurisdictions in battles of independence, roles and responsibilities. We then identified a series of key themes we were focused on such as data exchange, legal (cases and general), visa cancellation, deportation and AAT. We also coded for statistics, policy and practice. We imported all the reports and the readily available submissions to each inquiry into Nvivo and coded against these criteria. We then exported the information under each node to determine content on data exchange. In addition to this, we read through each public inquiry final report to confirm we had retrieved the most relevant information. For the policy documents, this information is available to the public via a subscription to LEGENDcom. We read through each of the policy instructions and determined which ones were relevant to s501 and its operation. We extracted key content on data exchange as it related to each agency.

In what follows, we critically analyse the development of data exchange practices over three specific periods—historical practice pre-2014, post-2014 to the present, and proposed future developments. This contextual analysis allows us to develop an understanding of how automation has been approached in the visa cancellation process over time and how the balance between human-non-human agents within data exchange networks has been struck. Crucially for this paper, it also allows us to trace practices before and after the introduction of automatic visa cancellation laws in 2014.

### Pre-2014 data exchange and visa cancellation—a historical overview

Our documentary analysis shows that there have been persistent concerns raised in government inquiries, extending at least as far back as the 1990s, about how potential non-citizens are identified for visa cancellation, and consistent calls for improvements to the development and coordination of standard procedures for collecting data on non-citizens. Standardisation across states and territories was also seen as an important priority by those leading independent reviews of the government’s administration of visa cancellation and deportation. In one of the first parliamentary inquiries that we examined from 1998, entitled the ‘*Deportation of Non-Citizen Criminals’,* the Joint Standing Committee on Migration found that cooperation between state/territory governments and the federal government department administering the ‘criminal deportation scheme’, was essential (Commonwealth of Australia 1998, p. xiii). This Inquiry took place after visa cancellation provisions in s501 had come into effect but had as its focus the discretionary deportation powers in section 200 of the *Migration Act 1958* (Cth). To contextualise the frequency of deportation at the time of the Inquiry, the department’s submission shows that there were 296 potential deportees as at 30 June 1997 (Department of Immigration and Multicultural Affairs, 1997, p. 8). Over a 6-year period from 1990 to 1996, 700 people were considered for deportation and 74% given warnings. In 1996/1997, 261 people were considered for deportation with 162 resulting in warnings, 92 deportation orders and 37 actual deportations (1997, p. 16). At the time, the department estimated that a further 100 people were not liable for deportation on account of living in Australia for 10 years prior to the offending (the ‘10-year rule’). Moreover, 300 people were not eligible because their imprisonment term was less than 12 months. Despite seemingly low numbers, especially when compared to visa cancellation today, the Inquiry was intently focused on the adequacy and efficiency of arrangements in place to remove convicted non-citizens.

The 1998 Inquiry has provided a window into the mode of data exchange that existed at the time and the prevailing views on its effectiveness, the latter being one of the terms of reference. While the Inquiry found that ‘[the department’s] effective management of the criminal deportation scheme’ had ‘generally been satisfactory’, they recommended that more formal agreements should be concluded between the department and state/territory governments to encompass how potential deportees are identified and when deportation hearings should occur (1998, p. xiv). The Joint Standing Committee on Migration recommended that standard procedures be developed to identify potential deportees held in prison and verify citizenship information provided by those in prison (recommendation 6), as well as clarify the extent of the department’s powers to gather information. A Memorandum of Understanding (MOU) between the department and state/territory governments setting out these formal arrangements was recommended to specifically ‘clarify the exchange of information under the *Migration Act 1958* (Cth)’. The Commonwealth Ombudsman (2006) would later repeat this recommendation for MOUs and the standardisation of procedures to identify those convicted and potentially liable for visa cancellation.

The mode of data exchange between states and territories and the federal department in charge of immigration in the late 1990s shows *ad hoc* arrangements regarding electronic data exchange. Data exchange relied upon prison lists in varying formats maintained by state/territory corrective service agencies and regional cooperative relationships with department offices. Concerned about a lack of efficiency in these arrangements, the department advised the Inquiry of their desire for electronic data exchange and a uniform approach, including a national register of all prisoners.

*We are hoping to be able to go to all of the justice and corrections ministries and be able to see whether they are willing to give it to us in a standard way... We are working on a request to states to be able to at least present it in, first, an electronic format, and second, an agreed format.* (1998, p. 52).

Table 1 demonstrates that data exchange practices varied considerably not only in terms of mode of cooperation, but also in terms of the accuracy of information and the frequency of provision. Across jurisdictions, the main consistency was around the primary role played by (most) prisons in identifying non-citizens who entered custody based on self-reporting, and then alerting regional offices of the department, through varying formats, ‘of the details of people born overseas’ (Department of Immigration and Multicultural Affairs, 1997, p. 3). Inconsistencies exist around whether those on remand are included and only NSW expressly indicated at the time that they supplied details of all people entering custody, regardless of the information supplied via self-reporting on citizenship status and/or country of birth.

**Table 1:** Data exchange as at 1997 (Department of Immigration and Multicultural Affairs, 1997, p.37).

State/territory	Mode of cooperation	Verification and details provided in data exchange
Western Australia	Monthly print outs of ‘all persons in prison who have indicated their place of birth is outside Australia’ are delivered to the department’s Perth office.	No other checks to verify information provided through self-reports are made. The printout contains the person’s name, date of birth, date of arrival in Australia, offence details, sentence and expected minimum term of incarceration.
New South Wales	Department of Corrective Services provides lists on a quarterly basis by computer disc using a Lotus123 spreadsheet of ‘all prisoners who enter the NSW gaol system for the period in question’ (p. 37).	The inmate must complete a Personal Description Form including questions about: identity, address, DOB, country of birth, nationality, date of arrival in Australia, Australian citizenship, possession and location of passport, all of which is not verified. Information provided to the Department includes full name, DOB, country of birth (as self-reported), date of arrival (as self-reported), whether a citizen, whether unsentenced, sentenced or on appeal, length of sentence and Master Index number.
Queensland	Monthly lists of ‘foreign nationals received’ based on self-reporting, provided to the department’s Brisbane office by QLD Corrective Services.	The list includes name, DOB, arrival year (as self-reported), country of birth, sentence length, offence, conviction date, place of conviction and release date.
South Australia	Regular lists on those sentenced to imprisonment for 12 months or more are retrieved by a computer program that runs a specific inquiry against the Justice Information System.	The list contains, name, alias, offence and act, address, sentence, date of sentence, DOB and country of birth.
Tasmania	Prison produces a list entitled ‘Persons Born Overseas who have been received into Prison’.	The department’s submission suggests no report for 16 months and delays on the next one. The information contains prisoner number, surname, given name, place of birth, DOB, gender, date received into prison, date discharged, reason for discharge.
Victoria	Computer printout is provided to the department’s Melbourne office by the Office of Corrections (VIC) ‘on all persons charged with a criminal act and who give their place of birth as outside Australia’ (p. 37). A second is provided if, and when, the person is subsequently convicted of the charge’ (p. 37).	The information includes prisoner name, DOB, place of birth, nationality, year of arrival in Australia (as self-reported), place of arrival, name of ship/airline, current location, number of prior convictions, court, remanded to (which court and date) offences and sentence. Quite a lot of information provided on the Victorian cohort and ahead of conviction.
Australian Capital Territory	ACT Attorney-General’s Department provides information. Unclear as to how this is provided and what it is based on.	The information contains the name, date of birth, nationality of persons convicted who are not Australian citizens. Not known if this information based on self-reporting or is verified.
Northern Territory	Quarterly list provided by NT Department of Correctional Services derived from a computer printout listing all prisoners in the NT.	The list contains name, DOB, marital status, nationality, sentence date, bond date, parole date, remand date.

The most common method at the time of finding out about citizenship status was self-reporting by those entering custody. This appears to align with practices implemented in the UK once automatic deportations were introduced in 2007, but was occurring in Australia much earlier under a discretionary cancellation and deportation regime. While critics of the UK system highlighted the racialized character of this approach (Bosworth, 2011; Kaufman, 2012), this possibility was not raised in the 1998 Inquiry although the ACT was concerned about the potential for deception when relying on self-reporting (1998, p. 52).

Various rub points around the cooperation between federal and state/territory jurisdictions were identified in the process of data exchange’ (1998, p. 14). Compliance with privacy legislation was a key concern of some governments. The Joint Standing Committee on Migration recommended that the department ‘clarify the legal position’ on its existing powers to increase efficiency and accuracy in accessing information on potential deportees and resolving any liability under privacy legislation for state and territory governments who supplied information to the department (1998, p. 60). These jurisdictional variations reveal an absence of a uniform national approach that might better lend itself to automation and the capacity to ‘flow like oil’. Instead, it demonstrates a reliance on human actors (including non-citizens) and *ad hoc* networks of data exchange to achieve accuracy and efficiency.

The 1998 Inquiry showed how the exchanged data is then analysed by way of human non-human actors in the decision-making process. At the time of this Inquiry, the department stated that information passed on in various formats was then entered into the department’s ‘criminal deportation computer data base’ (p. 17). Once the information on potential deportees was exchanged, the department assigned a case manager to conduct a ‘verification’ and establish ‘liability for deportation’ on a case-by-case basis by ‘verifying that the person is a non-citizen’ (1998, p. 15). The following electronic records were examined as part of the process: the citizenship database; movements database; department records; and penal records from states and territories (1998, p. 15). A deportation submission was then prepared for a decision-maker, which includes either a summary (deportation not recommended) or a comprehensive (deportation recommended) report. Overall, the system at that time reflected a high level of inter-agency data exchange continuing throughout a primarily human-mediated decision-making process. Before examining what we have been able to piece together regarding data exchange since the introduction of automatic visa cancellation in 2014, we turn to analyse a further development in the form of ‘computer-based decision-making’ in migration governance.

### Enabling computer-based decision making in migration law

Whereas the 1998 Inquiry showed minimal reliance on technology to produce lists and organise data, automation in decision-making came on the agenda a few years later in 2001. The *Migration Legislation Amendment (Electronic Transactions and Methods of Notification) Act 2001* (Cth) amended the *Migration Act 1958* (Cth) to pave the way for automated decision-making in migration control systems. Section 495A enabled the Minister to ‘arrange for use of computer programs to make decisions etc’. Upon introduction, clear guidance was provided to limit non-human decision-making, including the express statement that it not be used for visa cancellation for reasons explained below. The Minister for Immigration at the time of its introduction stated in the Second Reading Speech to parliament that the legislation ‘establishes a framework to allow for the use of computer programs to make decisions in the migration and citizenship context’ (p. 26528). The introduction of computer-based decision making in migration law was directly linked to the advent of its use in social security law, the home of Robodebt some years later. The allure of efficiencies made possible through automation is clearly a driving force behind this legislation with the Minister explaining that ‘computer based decision making will provide new opportunities for clients who have been previously restricted by office hours’, and thus the humans keeping them. Those submitting visa applications under the new system are promised ‘greater convenience’, with the safeguard that ‘visa and citizenship services will only be provided electronically after all security and integrity risks are satisfied’ (Commonwealth of Australia, House of Representatives, 2001, p. 26528). The greater reliance on information technology was seen as a flow-on from other initiatives in the department that have successfully utilised developments in technology to improve processes, such as electronic travel authorities. These establish a process to make travel to Australia easier for citizens from certain wealthy countries that have been assessed as low-risk and unlikely to be sending asylum seekers as tourists.

When introduced, the scope for computer based decision-making in migration was overtly circumscribed to preclude visa cancellation decisions. As the Second Reading Speech details:

*In the migration context, a computer program will only make decisions on certain visa applications where the grounds for grant are objective and where the criteria lend themselves to automated assessment.*

*A decision to cancel a visa will not be made by a computer program. Computer based processing is not suitable in these circumstances because these decisions require an assessment of discretionary factors.*

*Nonetheless, the legislative framework is sufficiently flexible to allow for technological advances which may occur in the future.*

*The challenge, however, is to have legislative strategies that allow for the use of these advances while providing adequate safeguards for both the integrity of government processes and achieving equity for clients* (Commonwealth of Australia, House of Representatives, 2001, p. 26529).

Visa cancellation decisions are overtly excluded from non-human decision making on account of the need to evaluate discretionary factors. However, the caveat is included here that developments in technology, such as the subsequent advent of generative AI, for example, might make this possible into the future. Before looking to the future, we trace developments in automation since the introduction of automatic visa cancellation in 2014 to the present.

### Data exchange and visa cancellation since the introduction of automatic visa cancellation—from 2014 to today

The arguments for standardised procedures for data exchange on those potentially liable to visa cancellation have increased in volume since 1998, yet *ad hoc* prisoner lists provided by states/territories appear to remain the norm. A 2016 Ombudsman report on the administration of s501 showed no MOUs had yet been agreed and informal arrangements persisted, concluding that the recommendation from its 2006 report was only partially implemented. It found that the process of going through prisoner lists to identify non-citizens liable to automatic visa cancellation was inefficient:

*The process of having to go through substantial prisoner lists is also time consuming especially given immigration records for many people who arrived before the 1980s are not fully computerised and may require the investigation of paper files* (Commonwealth Ombudsman, 2016, p. 10).

The Ombudsman concluded that the process was reactive and again recommended MOUs in its 2016 report (2016, p. 10). In response, the department noted the recommendation but did not agree to it in full. It argued that prison lists were sufficient and stated that the Australian Border Force (ABF), an operational arm of the department established in 2015 to focus on border controls, was ‘leading discussions with offices of state and territory corrections agencies to establish [MOUs] on services and information sharing’.^21^ It also said the Department’s preferred approach was to have ‘a broad headline agreement and tailored arrangements or specific topics sit under the agreement with letters of exchange’ (2016, p. 36). Our recent Freedom Of Information (FOI) inquiries on the current status of any MOUs on data exchange have found that there are still none in place.

In our analysis of parliamentary inquiries, we observed an increasing focus on efficiency and accuracy in identifying non-citizens who were potentially liable for visa cancellation and removal under discretionary provisions, not automatic visa cancellation. Proponents of this position seek to sharpen and widen the gaze beyond those caught by automatic visa cancellation provisions. One organisation with an intense focus on this cohort of non-citizens and driving the narrative around the need for improved detection of those potentially liable for visa cancellation under discretionary provisions was the Police Federation Association (PFA). The PFA called for the involvement of the National Criminal Intelligence System (NCIS) to link current systems (Commonwealth of Australia, 2017, p. 165). The Australian Criminal Intelligence Commission (ACIC) agreed that the intelligence systems currently operating were siloed and antiquated and that they were at the time engaged in a pilot scheme to build a new intelligence system (p. 166–8). The Joint standing Committee on Migration recommended that the Commonwealth fund the Australian Criminal Intelligence Commission to collect data ‘on the visa status of offenders for inclusion on their national database and the National Criminal Intelligence System’ (see Recommendation 14, p. 146). We sought to find out if this had occurred through a Freedom of Information request submitted to the ACIC in 2023, which returned no new agreement nationally to collate and identify convicted non-citizens using the NCIS.

This focus on the need for improved identification of those potentially liable for discretionary visa cancellation might suggest that the approach for automatic visa cancellations was satisfactory. In the department’s own assessment, they are identifying all those liable to automatic visa cancellation. According to the department’s 2018/2019 Annual Report (Commonwealth of Australia, 2019, p. 39), they met their target of identifying 100 per cent of those non-citizens subject to automatic visa cancellation:

*This outcome continues to reflect close collaboration with law enforcement agencies to identify non-citizens posing a risk to the community after they have been cleared to enter Australia. The Department and the ABF worked closely with state and territory correction services to identify visa holders serving custodial sentences in Australia and helped remove them at the end of their incarceration* (p. 39).

The department expressed confidence in a 2017 parliamentary inquiry into Migrant Settlement Outcomes, that avenues for identification for those liable to cancellation for discretionary or automatic visa cancellation were both adequate. The department argued that ‘[i]n circumstances where a non-citizen is engaged in antisocial or criminal behaviour, they will generally be referred to the Department for visa cancellation consideration’ (Department of Immigration and Border Protection, 2017, p. 18). The department stated that the establishment of the NCCC was aimed at centralising decision-making and referrals and building up expertise (2019a, 27 June 2018, p. 4). The introduction of the NCCC has created a focal point for the department to manage visa cancellation inquiries and decisions, but would appear to have had no demonstrable impact on the embrace, or use, of automation by the department.

In the 2017 Joint Standing Committee on Migration inquiry into Migrant Settlement Outcomes (paternalistically) entitled ‘No one teaches you to become an Australian’, as part of questions on notice, contemporary information was provided on how potential deportees are identified and referred from state/territory governments:

*The Department receives regular lists of non-citizens who have entered prisons, regardless of sentence. Generally, only those with a sentence of 12  months or more are recorded on departmental systems. All State and Territory corrective services agencies, except for the Australian Capital Territory, provide prison lists. Information sharing negotiations with the ACT Government are continuing* (DIBP Supplementary Submission 73, p. 6).

This indicates that prisons are still providing the lists of non-citizens and are thus determining or identifying who is a non-citizen, at least in the first instance. The continued reliance on prison lists to identify potential deportees was confirmed again in a public inquiry in 2019 entitled ‘Review processes associated with visa cancellations made on criminal grounds’ (2019a, Transcript 27 June 2018, p. 3), with no further information on how data transfer is effected. During a public hearing, the Assistant Secretary of the NCCC stated simply that the onus is on the department to identify potential deportees from prison lists provided by state and territory governments (2019a, Transcript 27 June 2018, p. 3). The report notes the human labour involved in effecting a decision:

*The Department was asked how mandatory cancellations are triggered, and it confirmed that it regularly receives lists of prisoners, goes through them and assesses liability for mandatory cancellation, then actions these cancellations* (p. 18).

No further information was provided as to how lists were scrutinised or examined and the extent of electronic data matching, or even if the lists were received electronically in a nationally consistent format. The latest Procedural Instruction entitled ‘Non-citizens in criminal detention’, a policy guide of the Government, stipulates that there are several ways in which the department is informed of non-citizens in prison, in addition to relationships with state/territory departments and prisons lists. The Procedural Instruction lists other sources of information including: ‘obtaining notices of convictions from courts; scanning law lists; requesting advice from parole officers or the probation service; scanning newspaper reports; and examining prison records’ (Department of Immigration and Border Protection, 2023a, p. 3).

Contemporaneously, the picture that emerges is of an automatic visa cancellation system that is not all that different from the system that existed prior to 2014. While neither sticky like glue, or flowing like oil, information is flowing. The department claims the system is 100 per cent effective in identifying those liable to automatic cancellation. The onus is on the department to identify non-citizens who are potentially liable from prison lists supplied by state/territory authorities. These are still, we presume, provided in varying formats and examined by human decision-makers on a case-by-case basis. We now turn to dig deeper into the legal framework for visa cancellation and interrogate the concept of ‘efficiency’ in this automatic visa cancellation system.

## How automatic is mandatory visa cancellation?

Our systematic search for automation in the automatic visa cancellation system has found no evidence of the use of sophisticated technology beyond the electronic sharing of data between state and federal agencies. However, the evidence uncovered has posed an even more fundamental, and unexpected, question about the nature of visa cancellation itself. So far, we have assumed an equivalence between the concepts of ‘automatic’ and ‘mandatory’ cancellation and have opted to use the term ‘automatic’ throughout this discussion, with its overtones of automation. However, the report of the 2019 Inquiry mentioned above included the intriguing observation that ‘While the cancellation is mandatory, it is not automatic’ (Commonwealth of Australia, 2019, p. 29). The legislation introducing this law reform refers to ‘mandatory visa cancellation’, although lawyers and various agencies use ‘mandatory visa cancellation’ and ‘automatic visa cancellation’ interchangeably. We therefore dug deeper into the documentary evidence to try to determine what being mandatory, but not automatic, might mean.

We discovered that the introduction of mandatory visa cancellation to cancel a visa without natural justice or prior notice to the visa holder, was accompanied by a process through which a visa holder could seek ‘revocation’ of the visa cancellation decision. Section 501CA of the *Migration Act 1958* (Cth) stipulates that as soon as practicable after making the decision to cancel a visa under s501(3A), the person must be given a written notice that sets out the original decision, and the particulars of information relevant to the decision, and invites them to apply for revocation. Section 501CA(4) gives the Minister or a delegate the power to revoke a decision to cancel a visa under s501(3A), if the person makes representations in accordance with the invitation and the Minister or delegate is satisfied that the person passes the character test (as defined by s501 and including all limbs) or there is another reason why the original decision should be revoked. Delegates must be guided by Ministerial Direction no. 99 when making a revocation decision under s501CA(4). The Minister is not bound by the Direction nor required to table a notice in Parliament of a decision under s501CA (Department of Immigration and Border Protection, 2023b, p. 17). In effect, mandatory visa cancellation is not automatic if you apply for revocation.

Approximately three quarters of those who have their visa cancelled do apply for revocation of the mandatory visa cancellation decision. From 2015 to 31 January 2021, 5,921 mandatory visa cancellations under s501(3A) were issued and 4,557 people applied for revocation, or just over 75 per cent (Department of Home Affairs, 2021). During this same time period, 34 applications were deemed invalid or non-compliant. Revocation must be applied for within 28 days and there is no statutory basis for an extension. Applying for revocation within this tight deadline is challenging given that people are in prison or immigration detention where mail and legal advice can be difficult to access in a timely way. Revocation outcomes take a long time for the department to decide, and that length of time is increasing. In 2015, the time from mandatory visa cancellation to a revocation outcome took 149 days on average, and in 2022 the average time was 641 days (Department of Home Affairs, 2022).

In summary, just under a quarter of those who receive a mandatory visa cancellation do not apply for revocation, or their application is invalid, and therefore their mandatory visa cancellation becomes automatic after the revocation deadline passes. The overwhelming majority apply for revocation and await a long process to receive an outcome, almost always while being detained in immigration detention. This is how the system was designed, with the Minister stating when the Bill was introduced to parliament that this process ‘will deliver the key benefit of providing a greater opportunity to ensure noncitizens who pose a risk to the community will remain in either criminal or immigration detention until they are removed or their immigration status is otherwise resolved’ (Commonwealth of Australia, House of Representatives, 2014, p. 10327). The goal of community protection was explicitly elevated over both efficiency or procedural fairness. A portion of people opt to be removed from immigration detention before their revocation outcome is decided. Five percent of those removed from 11 December 2014 to 31 December 2019 received favourable revocation decisions after being removed from Australia (Department of Home Affairs, 2020).

Revocation is the point at which individuals move from being unlawful persons dealt with collectively in a mandatory visa cancellation machine, to an enlivened process where their individual circumstances are considered (albeit under restrictive Ministerial Directions), and many are successful. Between 1 January 2017 and 31 December 2018, 38 per cent of revocation requests were successful and took an average of 305 days (Department of Home Affairs, 2019b). The revocation process, if applied for, provides a shift to a new realm involving human actors empowered to act with some discretion (see O’Malley, 2010), with the result that at least a third of mandatory visa cancellation decisions are overturned.

For those who are unsuccessful in their application for revocation, a merits review and judicial review process is available in limited circumstances. Where a delegate makes the decision not to revoke a mandatory visa cancellation, a review by the Administrative Appeals Tribunal (AAT) must be applied for within 9 days of a revocation outcome decision. It is not possible to extend this timeframe, thus those who miss this deadline will again experience mandatory visa cancellation as automatic, albeit factoring in the delay while awaiting the revocation outcome in the first place. At the AAT stage, on average about 20 per cent of the department’s decisions are overturned (2019a, vii). In AAT visa cancellation cases around 50 percent of applicants are represented by a lawyer or migration agent (2019a, p. 44), a factor that can potentially increase the chances of achieving individualised justice. The Minister can still set aside a decision of the AAT not to cancel a visa if it is in the national interest (s501BA). Between 11 December 2014 and 31 May 2018, the Minister has used the power to overrule the AAT decision in a mandatory revocation case 13 times (s501BA). Finally, there is a pathway for judicial review of mandatory visa cancellation cases and of the 124 delegate decisions affirmed by the AAT between 1 July 2017 and 31 March 2018, 37 non-citizens went on to seek judicial review (2019a, p. 17).

**Figure 2:**
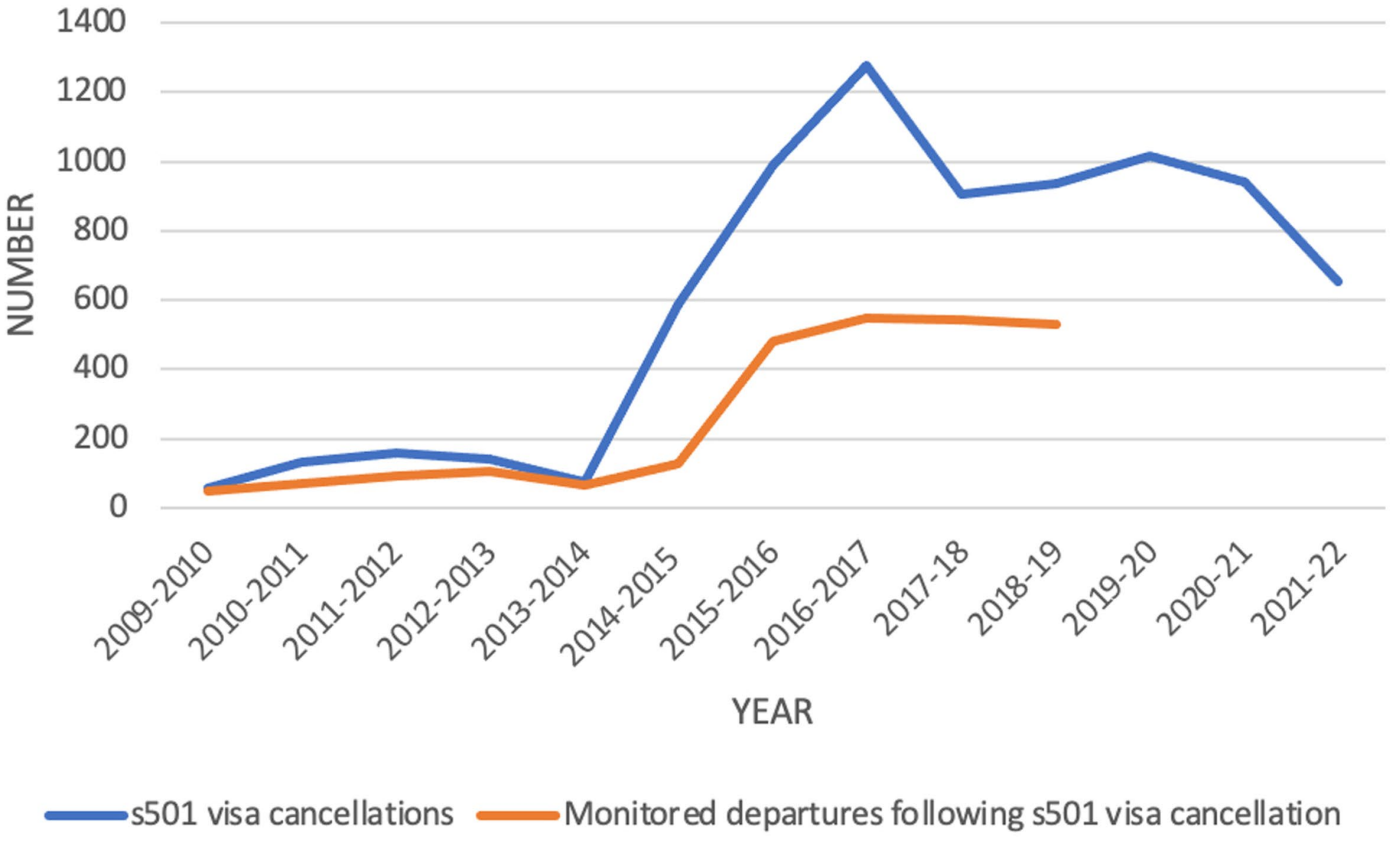
s501 visa cancellations and monitored departures following s501 visa cancellations 2009/10–2021/22.

The introduction of automatic visa cancellation in 2014 did not completely remove discretion from the decision-making process, but rather displaced consideration of natural justice and individual factors until the revocation and review phases for those visa holders willing and able to lodge these challenges. Rather than increase the efficiency of the removal machinery, the legislative change has introduced additional sources of delay and prolonged detention for individuals who would not have had their visas cancelled in the first place, had natural justice been applied from the outset. Mandatory cancellation, it transpires, is not only not very automated, it is also not wholly automatic. As Figure 2 reveals, many cancellations (the vast majority of them using mandatory provisions) do not lead to removal, with the high level of revocations and decisions to set aside accounting for much of the discrepancy.

## Looking ahead—automation and visa cancellation

We have shown so far that advanced forms of automation have been either lacking or explicitly rejected within the s501 visa cancellation system. However, noting the increased use of automation over the last decade by other government departments, recent attempts by the department of immigration to outsource visa and citizenship services and enhance technology raise the spectre of greater capabilities for computer-based decision making in the future. Australian governments have shown a voracious appetite for new and automated technological systems or ‘enhancements’ that claim to offer efficiencies, without rigorous exploration of how and what they are producing. In 2017, the department issued a tender for ‘a new visa service delivery business, including new technologies to help design and build a global digital visa-processing platform’ (Commonwealth of Australia, 2020, p. 19). Critics of outsourcing referenced the UK experience where the new visa processing system has led to ‘price-gouging of visa applicants, vulnerable applicants being exploited and private providers using long delays in processing to drive interest in a quicker, premium, high-priced product’ (2020, p. 27). A parliamentary committee recommended against the tender proceeding, and it was subsequently terminated by the Minister responsible in 2020. While this process concerns the granting of visas, another arm of migration control, it reminds us that we cannot be complacent about the potential for automation to expand to visa cancellation at a later point in time. When computer-based decision-making was introduced in 2001, visa cancellation decisions were overtly excluded from non-human decisions, but as technological capability improves this may not remain the case. The Robodebt Royal Commission’s recommendation for a body to monitor and audit automated decision-making processes would go some way to safeguarding against any bias and injustice within migration control systems.

The UK’s experience of relying on technological solutions and their belief in infallibility in other areas, such as the subpostmaster’s case, reveals the potential harms of a naïve approach to automated decision-making (McGuire and Renaud, 2023). The subpostmaster’s case involved the introduction by the Post Office of a new and supposedly more efficient accounting system across its local network that subsequently identified shortfalls and accused local offices of theft. Over 700 people were prosecuted and some served sentences of imprisonment before a judge ruled the prosecutions unsound due to their reliance on software that was mistaken. The case represents a striking case of miscarriage of justice and alerts us to the need to look inside the ‘blackbox’ to understand what is being decided by computers and how that evidence is produced.

The lack of clarity behind Australia’s uptake of automation in visa decisions has drawn criticism. While the presence of discretionary factors made computer-based decision-making unsuitable when initially introduced in 2001, some visa decisions involving discretionary factors have succumbed to automation. Lawyers argue decisions involving discretionary factors are being decided by computer-based programs (Law Council of Australia, 2022). For example, in determining a Working Holiday visa, a visa class where autogrants are made, ‘some kind of subjective assessment of an applicant’s travel intentions’ is required (2022, p. 26). The department released information pursuant to an FOI request in 2021 which confirmed that the proportion of visa decisions made through automation had increased from 66% in 2010/2011 to 73% in 2020/2021. The department also stated that its systems ‘cannot, by design, issue refusal decisions’, implying that the granting of visas is the only aspect which is, at this stage, automated (2022, p. 65). Any changes to what can be decided by computer-based programs does not require parliamentary scrutiny (2022, p. 58), underscoring the need for clarity and transparency. How these programs are pre-loaded, and with what assumptions, are also critical questions that demand an answer.

The Law Council recommends that clear authorisation to use automated decision-making or AI in making decisions should be introduced to clear up any uncertainty and to be transparent about who is making a decision and exercising statutory powers. This is an important consideration if ‘blackboxing’, discussed earlier, is to be avoided. The Commonwealth Ombudsman produced a ‘Better Practice Guide’ for automated decision-making in 2007 and an updated version in 2019. The tool is designed to be practical and includes a checklist for use in design and implementation of automated systems. It emphasises ongoing quality assurance processes. The principles cover ethics, discretion, privacy, administrative law, governance, transparency and accountability, and monitoring and evaluation (Commonwealth Ombudsman, 2019).

In an area of law where people can be declared unlawful and held in immigration detention and/or later removed from Australia, a lack of clarity and accuracy in using technology has major consequences for individuals and for community safety generally. A series of Commonwealth Ombudsman reports have identified that Australian citizens, and non-citizens, have been unlawfully held in immigration detention because decision-makers from the department have relied on partial information systems that fail to consider historical records and information available through online systems. Critically, the Commonwealth Ombudsman highlighted that these were not new problems, repeating errors of over a decade ago (Commonwealth Ombudsman, 2018a, p. 3; Commonwealth Ombudsman, 2018b).

## Conclusion: Robodeport or surveillance fantasy?

Mandatory visa cancellation was introduced with much fanfare to ensure ‘that noncitizens do not pose a risk to the Australian community’ (Commonwealth of Australia, House of Representatives, 2014, p. 10327). While no explicit mention was made of the introduction of new technologies to support the new system, the emphasis on automatic cancellation created an illusion of enhanced technocratic efficiency. However, rather than uncovering Robodeport, we found the spectre of a highly efficient and *automated* machinery producing *automatic* visa cancellations to be nothing more than a ‘surveillance fantasy’. Our systematic documentary analysis revealed the use of digital technology within the s501 system to be minimal and extremely basic in comparison with other migration control functions operating in Australia, being reliant on voluntary electronic data exchange between state and federal authorities with no automated data matching or algorithmic decision-making. Yet our analysis has revealed that systems based on less sophisticated forms of information handling and reliant on human decision-making nevertheless continue to raise age-old questions of governance concerning the appropriate balance to be struck between efficiency, accuracy and procedural fairness.

Although driven largely by human actors, this ‘surveillance fantasy’ nevertheless, is responsible for systemic and ongoing harms that potentially stratify the border for racialised and criminalised populations. This system is designed to keep a tight grip on non-citizens characterised as a risk to the Australian community and is not incentivised to be efficient. The post-2014 system introduced long delays in immigration detention while awaiting the application of natural justice considerations which, however limited in scope, would previously have been applied before cancellation. In fact, the lack of technology in the process potentially exacerbates delays, as human actors are required to mediate disconnected databases and prescriptive decision-making guides that serve to distance decision-makers from those directly impacted. Accuracy is claimed by the Department, for example through individualised cross-checking of data, but unlawful detentions are still occurring. The fact that we have been forced to put together this analysis through a painstaking process of sifting through many parliamentary inquiries, independent reports and policy analyses plus the lodgment of multiple freedom of information requests also indicates that the system is far from transparent.

In sum, our exploration of the automatic visa cancellation process has found that human decision-making systems can also operate inside a figurative ‘black box’ and produce unjust outcomes where they are governed by rigid policies that reduce the space for individualised justice. In this case it is the *automatic* nature of the visa cancellation system, rather than its *automation*, that is producing injustice for non-citizens who have come into conflict with the criminal law. That said, any future introduction of automated processes, without changes in the existing legal framework, is likely to merely entrench existing biases and produce further injustices. At an even more fundamental level, the stratification of convicted persons on the basis of citizenship which underpins the s501 system, reveals the centrality of formal citizenship in a society willing to engage in violent re-bordering projects such as automatic visa cancellation in order to more clearly delineate the boundaries of membership and belonging.

## Data availability statement

The data supporting the conclusions of this article are publicly available from the sources cited.

## Author contributions

LW: Conceptualization, Funding acquisition, Project Administration, Writing – original draft, Writing – review & editing. AG: Formal Analysis, Funding acquisition, Project Administration, Writing – original draft, Writing – review & editing.

## Footnotes

^1^At law, the ‘deportation’ power is contained within s200 of the *Migration Act 1958* (Cth). This has been seldom used since the creation of visa cancellation powers involving the character test in s501 of the *Migration Act 1958* (Cth). There are also general visa cancellation powers in s116 of the *Migration Act 1958* (Cth).

^2^In the years immediately prior to this legislation being enacted, and for a 5  year period afterwards, the proportion of visa cancellations arising from the ‘substantial criminal record’ provisions remained fairly steady at between 93 and 97%. Source: Freedom of Information request to Department of Home Affairs (2019a) FA 19/12/01189 s501 cancellations by (legislative) ground from 1 July 2012 to 30 June 2019.

^3^The mandatory cancellation provisions were controversially introduced by the *Migration Amendment (Character and General Visa Cancellation) Act 2014* (Cth).

^4^Since the name of the relevant department—now contained within the Department of Home Affairs—has changed several times over the period covered by this article, generic terminology such as ‘the department’ or ‘immigration authorities’ will be used throughout.

^5^Note that the term ‘mandatory’ is not used in the 2014 amending legislation, which states that the Minister ‘must’ cancel a visa if the statutory requirements are met. The Second Reading Speech of the Minister when introducing the Bill to parliament, and the Explanatory Memorandum, use the terminology of ‘mandatory cancellation’.

^6^‘Crimmigration’, a term now in widespread use, was coined in 2006 by Juliet Stumpf to refer to the merging of criminal and migration law and practice.

^7^Australia includes two territories—the Australian Capital Territory and the Northern Territory which have a different relationship with the federal government than the fully fledged states.

^8^For example, the Australian Taxation Office participates in electronic data exchange and data matching processes with immigration authorities with a view to identifying tax debts incurred by non-citizens, while hospitals my wish to identity patients who are not entitled to government-funded care.

^9^A Guardian newspaper article in April 2023 reported that the British Home Office was assessing whether AI could be used to make inroads into the 138,052 asylum cases awaiting adjudication (Gentleman, 2023).

^10^Note that criminal courts in Australia have generally not been involved in deportation decisions.

^11^Migration Amendment (Strengthening the Character Test) Bill 2018; 2019; 2021.

^12^This approach has some superficial similarities with the ‘aggravated felonies’ measures enacted in the US from the late 1980s (Stumpf, 2006, p. 383).

^13^Migration Amendment (Strengthening the Character Test) Bill 2021 Explanatory Memorandum.

^14^Alex Hawke, MP, Second Reading Speech, Migration Amendment (Strengthening the Character Test) Bill 2021, House of Representatives, 16 Feb 2022.

^15^At the time of the ‘migration policing networks’ research, immigration authorities had just begun to embed liaison officers in some local courts in an attempt to identify potentially removable non-citizens but there appeared to be no systems of electronic data exchange in place (Weber, 2013, p. 116–17).

^16^Known at different stages in its development as the PAYG Manual Compliance Intervention Program, Online Compliance Intervention, Employment Income Confirmation and Check and Update Past Income.

^17^Commonwealth of Australia (2022): Royal Commission into the Robodebt Scheme (Volume 1). Canberra: Commonwealth of Australia from Royal Commission into the Robodebt Scheme—Full Report.

^18^Funded by an Australian Research Council Discovery Project grant DP210100931. Note that the term ‘criminal deportation’ does not exist in law but is our operational definition for visa cancellations under s501 of the Migration Act 1958 (Cth) followed by administrative removal.

^19^In Australia, a Freedom of Information (FOI) request enables a person to access information held by government, including policies and decisions, subject to a number of exceptions. Access to information at a federal level is governed by the *Freedom of Information Act 1982* (Cth).

^20^Commonwealth of Australia (2021) *Migration and Citizenship Legislation Amendment (Strengthening Information Provisions) Bill 2020* (Australian Senate Legal and Constitutional Affairs Committee). Retrieved from: https://www.aph.gov.au/Parliamentary_Business/Committees/Senate/Legal_and_Constitutional_Affairs/StrengtheningInformation/Report; Commonwealth of Australia (2019) *Migration Amendment (Strengthening the Character Test) Bill 2018* (Australian Senate Legal and Constitutional Affairs Committee). Retrieved from: https://www.aph.gov.au/Parliamentary_Business/Committees/Senate/Legal_and_Constitutional_Affairs/Charactertest2019/Report; Commonwealth of Australia (2018) *Migration Amendment (Strengthening the Character Test) Bill 2018* (Australian Senate Legal and Constitutional Affairs Committee). Retrieved from: https://www.aph.gov.au/Parliamentary_Business/Committees/Senate/Legal_and_Constitutional_Affairs/CharacterTest/Report; Commonwealth of Australia (1998) *Inquiry into the Deportation of non-citizen Criminals* (Joint Standing Committee on Migration). Retrieved from: https://www.aph.gov.au/~/media/wopapub/house/committee/mig/report/criminal_deportation/criminaldeportation_pdf.ashx; Commonwealth of Australia (2019) *Review processes associated with visa cancellations made on criminal grounds* (Joint Standing Committee on Migration). Retrieved from: https://www.aph.gov.au/Parliamentary_Business/Committees/Joint/Migration/Visacancellationprocess/Report; Commonwealth of Australia (2017) *Inquiry into Migrant Settlement Outcomes* (Joint Standing Committee on Migration). Retrieved from: https://www.aph.gov.au/Parliamentary_Business/Committees/Joint/Migration/settlementoutcomes/Report.

^21^The Australian Border Force was created in 2015 through the merger of the Australian Customs and Border Protection Service and the Department of Immigration and Border Protection. The ABF was then moved into the Department of Home Affairs when it was created as a mega-Department in 2017.
